# Transition metal trifluoroacetates (M = Fe, Co, Mn) as precursors for uniform colloidal metal difluoride and phosphide nanoparticles

**DOI:** 10.1038/s41598-019-43018-8

**Published:** 2019-04-29

**Authors:** Christoph P. Guntlin, Kostiantyn V. Kravchyk, Rolf Erni, Maksym V. Kovalenko

**Affiliations:** 1 Department of Chemistry and Applied Biosciences, ETH Zürich - Laboratory for Inorganic Chemistry, Vladimir Prelog Weg 1, CH-8093 Zürich, Switzerland; 2 Laboratory for Thin Films and Photovoltaics, Empa – Swiss Federal Laboratories for Materials Science and Technology, Überlandstrasse 129, CH-8600 Dübendorf, Switzerland; 3 Electron Microscopy Center, Empa – Swiss Federal Laboratories for Materials Science and Technology, Überlandstrasse 129, CH-8600 Dübendorf, Switzerland

**Keywords:** Nanoparticle synthesis, Energy

## Abstract

We report a simple one-pot synthesis of uniform transition metal difluoride MF_2_ (M = Fe, Mn, Co) nanorods based on transition metal trifluoroacetates (TMTFAs) as single-source precursors. The synthesis of metal fluorides is based on the thermolysis of TMTFAs at 250–320 °C in trioctylphosphine/trioctylphosphine oxide solvent mixtures. The FeF_2_ nanorods were converted into FeF_3_ nanorods by reaction with gaseous fluorine. The TMTFA precursors are also found to be suitable for the synthesis of colloidal transition metal phosphides. Specifically, we report that the thermolysis of a cobalt trifluoroacetate complex in trioctylphosphine as both the solvent and the phosphorus source can yield 20 nm long cobalt phosphide nanorods or, 3 nm large cobalt phosphide nanoparticles. We also assess electrochemical lithiation/de-lithiation of the obtained FeF_2_ and FeF_3_ nanomaterials.

## Introduction

Synthesis of nanoscale inorganic materials remains an active research area in inorganic chemistry, owing to the unique and improved material properties that emerge with respect to their bulk counterparts^1–7^. Downsizing is particularly important for conversion-type cathode materials for Li-ion batteries such as transition metal fluorides. These compounds offer one of the highest capacities among conventional cathode materials (e.g., 571 mAh g^−1^ for FeF_2,_ 237 mAh g^−1^ for FeF_3_ *vs*. 148 mAh g^−1^ for LiCoO_2_)^8,9^, but suffer from reduced rate capabilities and cycling stabilities, which are associated with low electronic conductivity and considerable structural reconstruction of the electrodes during cycling^10–16^. In this context, reducing the primary grain size of materials has become the main strategy for reducing polarization and improving the overall kinetics of Li^+^ insertion. Nanosized materials have a considerably shorter in-solid diffusion path with less mechanical stress during phase conversion.

The synthesis of uniform transition metal difluoride nanocrystals (NCs) and nanoparticles (NPs) is generally complicated by the highly reactive and hazardous nature of commonly used fluorine sources, e.g., F_2_^17^, HF^18^, and NH_4_F^19,20^. Several liquid-phase chemical approaches, including co-precipitation^19–23^, hydrothermal^24–27^, and solvothermal^28,29^ syntheses, have been used to synthesize transition metal fluorides. However, these methods yield NCs of irregular shape and broad size distributions. In 2005, an alternative approach was reported by Yan *et al*.^30^ for the synthesis of LaF_3_ nanoparticles (NPs) based on thermolysis of a single-source lanthanum trifluoroacetate precursor in high-boiling point organic solvents. The proposed surfactant-assisted synthetic pathway enabled control over nucleation and growth of LaF_3_ NPs by adjusting the capping ligands and the reaction temperature. In subsequent studies, a variety of different colloidal NPs, including NaYF_4_^31^ and GdF_3_^32^, were reported by Murray *et al*. based on single-source precursors. The reports of Yan *et al*.^30,33,34^, Murray *et al*.^31,32^ and others^35–40^ have guided this work, wherein we report on applications of transition metal (Fe, Mn, Co) trifluoroacetate complexes (TMTFAs) as precursors for the synthesis of uniform colloidal NPs of Fe, Mn, and Co difluorides. In these precursors, both transition metal and fluorine are integrated into one compound providing a high control over the size of transition metal (Fe, Mn, Co) fluoride NPs.

We achieved a colloidal synthesis of highly uniform Fe, Mn, and Co difluoride nanorods (NRs) through thermolysis of inexpensive TMTFAs in the solvents trioctylphosphine (TOP) and trioctylphosphine oxide (TOPO). Control over the size and shape of these nanomaterials was achieved by adjusting the temperature and decomposition time and by the addition of oleic acid (OA) as a long-chain surface ligand. In addition, we show that CoF_2_ NRs can themselves serve as precursors for nanomaterial synthesis, yielding 20 nm long cobalt phosphide NRs or amorphous, 3 nm large cobalt phosphide NPs upon reaction with TOP. Furthermore, we used fluorine gas to synthesize FeF_3_ NRs by fluorination of FeF_2_ NRs. Finally, we present investigations of electrochemical lithiation/de-lithiation in the synthesized FeF_2_ and FeF_3_ NRs.

## Experimental Section

### Chemicals

FeCl_3_ anhydrous (Alfa Aeser, 98%, 12357), CoCl_2_ anhydrous (Sigma-Aldrich, ≥98.0% 60818), MnCl_2_ anhydrous (Alfa Aeser, 99%, 12315), trifluoroacetic acid (TFA, Fischer, T/3255/PB05, 100 mL), trioctylphosphine (TOP, Strem, 15–6655, >97%), trioctylphosphine oxide (TOPO, Strem, 15–6661, 99%), oleic acid (OA, Sigma-Aldrich, 364525), ethanol (Merck, 1.00983.1011), and toluene (Sigma-Aldrich, 34866).

### Synthesis of Fe trifluoroacetate complex

[denoted as a “Fe_3_OTFA”]. “Fe_3_OTFA” was synthesized according to previous reports^41^. The chemical composition of “Fe_3_OTFA” precursor corresponds to the following formula: [Fe_3_(μ_3_-O)(CF_3_COO)(μ-CF_3_COO)_6_(H_2_O)_2_]·CF_3_COOH.

### Synthesis of Co trifluoroacetate complex

[denoted as an “Co(TFA)_2_”]. CoCl_2_ (10 g, 0.077 moles) was mixed with trifluoroacetic acid (100 mL, 1.307 mol) in a 250 mL two-neck flask equipped with a double-mantled 30 cm long Dimroth cooler. The reaction mixture was refluxed at 95 °C for 3.5 days under nitrogen flow. The resulting blue solution was cooled to 50 °C and 100 mL of dried toluene was added to precipitate “Co(TFA)_2_”. The product was filtrated under a N_2_ atmosphere following by a washing step with dried toluene (30 mL) and drying under vacuum for 24 h. The product is highly hygroscopic. Its crystal structure is unknown (powder XRD pattern of the “Co(TFA)_2_” precursor is shown on Figure S1).

### Synthesis of Mn trifluoroacetate complex

[denoted as an “Mn(TFA)_2_”]. “Mn(TFA)_2_” was synthesized according to previous reports^41^. The chemical composition of the “Mn(TFA)_2_” precursor corresponds to the following formula: Mn_2_(CF_3_COO)_4_(CF_3_COOH)_4_.

### Synthesis of FeF_2_ NRs

“Fe_3_OTFA” (562 mg, 0.5 mmol), was mixed with TOP (10 mL, 22.4 mmol) and OA (0–0.952 mL, 0–3 mmol) in a 50 mL three-neck flask. Afterward, the reaction mixture was dried under vacuum at 110 °C for 1.5 h followed by heating to 320 °C at a heating rate of 6–18 °C min^−1^. Finally, the reaction was quenched to 200 °C with compressed air followed by cooling in an ice-water bath with concomitant injection of anhydrous toluene (20 mL) into the crude solution at approximately 120 °C. Different amounts of OA and different heating rates controlled the length of the FeF_2_ NRs (see SI for details, Table S1). The FeF_2_ NRs were washed two times with a toluene/ethanol mixture and separated by centrifugation. After the second washing step, the NRs were redispersed in toluene (2–4 mL) and stored under ambient conditions.

### Synthesis of MnF_2_ NRs/NPs

Colorless “Mn(TFA)_2_” powder (509 mg, 0.5 mmol) was mixed with TOPO (8.8 g, 23 mmol) or TOP (10 mL, 22 mmol) in a 50 mL three-neck flask under a N_2_ flow to obtain short (15–20 nm) or long (25–35 nm) MnF_2_ NRs, respectively. Then, the reaction mixture was dried under vacuum at 110 °C for 1.5 h followed by slow heating (6 °C min^−1^) to 250 °C under a N_2_ flow (see SI for details, Table S2). Afterward, the reaction mixture was quenched following the washing procedure as described above for FeF_2_ NRs.

### Synthesis of CoF_2_ NRs/NPs

“Co(TFA)_2_” (0.5 g) was mixed with TOPO (8.8 g, 23 mmol) and OA (0.5 mL, 1.6 mmol) in a 50 mL three-neck flask under N_2_ flow. The reaction mixture was then dried under vacuum at 110 °C for 1.5 h under stirring (1400 rpm) followed by slow heating (6 °C min^−1^) to 300 °C (see SI for details, Table S3). Afterward, the temperature was maintained for 20 min followed by the quenching and washing steps as described above for the FeF_2_ NRs.

### Synthesis of Co_2_P NPs and Co_2_P NRs

In a typical synthesis of ~3 nm Co_2_P NPs, “Co(TFA)_2_” (0.5 g), TOP (10 mL, 22 mmol), and OA (0.5 mL, 1.6 mmol) were loaded into a 50 mL three-neck flask and dried under vacuum at 110 °C for 1.5 h. The reaction mixture was slowly heated to 300 °C at a heating rate of 6 °C min^−1^ and maintained at this temperature for 1.5 h. The synthesis of Co_2_P NRs were performed in the same was as that of the Co_2_P NPs; however, OA was added to the reaction mixtures and the heat-treatment time was prolonged to 2 h. Afterward, the reaction mixtures of Co_2_P NPs and Co_2_P NRs were quenched followed by the washing procedure, as described above for the FeF_2_ NRs.

### Synthesis of FeF_3_ NRs

In a typical synthesis of FeF_3_ NRs, a ~150 mg portion of the 120 nm long FeF_2_ NRs in powder form was placed in a closed Al_2_O_3_ tube and Al_2_O_3_ crucible as a container. The tube was then dried by applying a vacuum for 10 min followed by purging with N_2_ for 20 min at room temperature. Under an N_2_ flow, the sample was heated in a tube furnace (Across International, STF1200) to 500 °C. At this temperature, a flow of F_2_/Ar (Linde, 9.9% of F_2_ in Ar) gas was set for 10 min. Then, an N_2_ purge was applied for 30 min, and the tube was cooled down to room temperature. The obtained FeF_3_ NRs were stored under vacuum. A schematic diagram of the oven set-up is shown in Figure S2.

### Powder XRD

Powder diffraction patterns of FeF_2_ NRs, MnF_2_ NRs, and Co_2_P NRs and Co_2_P NPs were obtained in transmission mode on a Stoe STADI P powder X-ray diffractometer (Cu Kα_1_ radiation, λ = 1.540598 Å, germanium monochromator). For CoF_2_ NRs, the patterns were acquired on an STOE IPDS II single crystal diffractometer (image plate detector, a sealed tube with Cu Kα radiation, λ = 1.54186 Å, graphite monochromator, monocap-collimator).

### HR-, TEM, and SAED

High-resolution (HR) and low-resolution transmission electron microscope (TEM) images and selected area electron diffraction (SAED) patterns were taken with a JEOL JEM-2200FS microscope operating at 200 kV. EDX maps in scanning transmission electron microscopy mode were recorded on a probe aberration-corrected FEI Titan Themis operated at 300 kV using a SuperEDX detector with a beam current of about 1 nA. EDX mapping was stopped when degradation of the particles due to radiation damage was observed. Samples for TEM analysis were prepared on carbon-coated Cu grids (Ted-Pella).

### EDX

The energy-dispersive x-ray (EDX) spectroscopy measurements were performed on a nanoSEM 230 (FEI).

### Electrochemical characterization of FeF_2_, and FeF_3_ NRs

FeF_2_ electrodes were prepared by mixing FeF_2_ NRs (58%), carbon black (CB, 21%), graphene oxide (GO, 13%), and poly(vinylidene) fluoride binder (pVdF, 8%). First, the ~120 nm long FeF_2_ NRs were mixed with CB in a ball mill (Pulverisette 7, Fritsch, ZrO_2_ balls and beaker) for 1 h at 300 rpm followed by a heat treatment at 500 °C for 0.5 h under a N_2_ atmosphere to remove the ligands and to improve the carbon embedding. Then, the FeF_2_/CB powder was ball-milled with pre-milled GO (synthesized by the Brodie method^42^), pVdF, and *n*-methyl-2-pyrrolidone (NMP) for 2 h, at 500 rpm. The final slurry was brushed directly on Al foil and dried under a vacuum at 80 °C for at least 24 h. FeF_3_ electrodes were prepared following the procedure described above for FeF_2_ electrodes but without the addition of GO. The composition of the FeF_3_ slurry was 58% of FeF_3_ NRs, 34% of CB, and 8% of pVdF. Coin-type cells were assembled in a glovebox with the use of a one-layer glass fiber (Whatman) separator and 1 M LiPF_6_ in ethylene carbonate/dimethyl carbonate (1/1 by wt.) electrolyte (300 µL per cell). Li metal served as both a reference and counter electrode. Electrochemical measurements were performed on an MPG2 multi-channel workstation (BioLogic). Prior to electrochemical cycling, the FeF_2_ and FeF_3_ electrodes were tested by cyclic voltammetry at scan rates of 0.2 and 0.1 mV s^−1^ for 5 and 2 cycles, respectively. For galvanostatic measurements of the FeF_2_ electrodes, constant current-constant voltage (CCCV) mode was applied for discharge and charge steps at 1.5 and 4.0 V *vs*. Li^+^/Li. The constant voltage was maintained until the measured current was equal to 1/5 of the initial current value. The obtained capacities were normalized to the mass of the FeF_2_ or FeF_3_ NRs.

## Results and Discussion

### Synthesis of transition metal (Fe, Co, Mn) difluoride NPs

**Figure 1:**
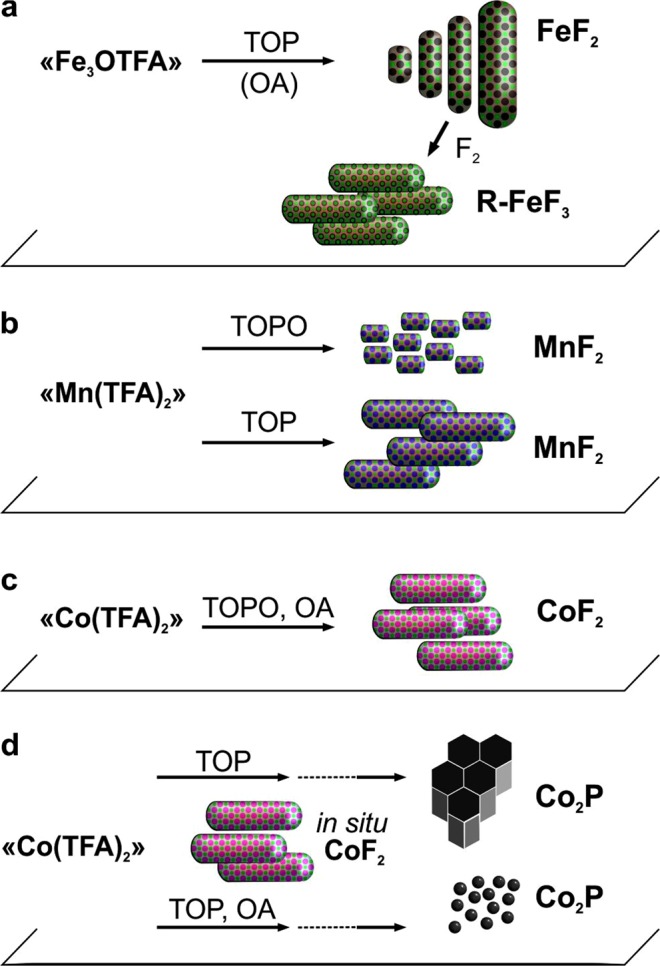
Schematic illustration of the synthesis of FeF_2_ and FeF_3_ (**a**), MnF_2_ (**b**), CoF_2_ (**c**), and Co_2_P NRs and Co_2_P NPs (**d**) with corresponding metal trifluoroacetate complexes [“Fe_3_OTFA”, “Mn(TFA)_2_” and “Co(TFA)_2_”].

**Figure 2:**
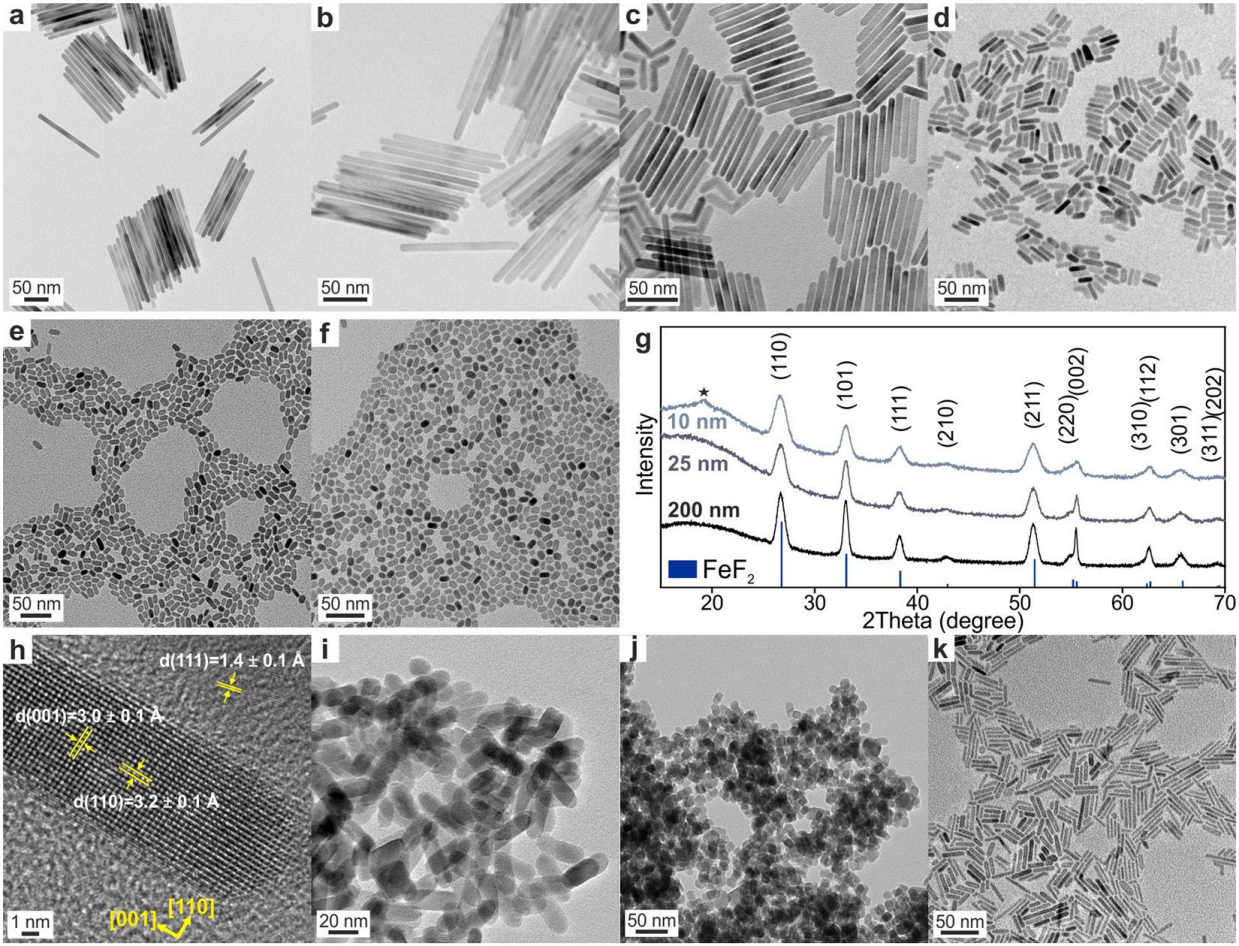
Characterization of MF_2_ (M = Fe, Co, Mn) NRs. (**a**–**f**) TEM images of ~200 nm (**a**) ~120 nm, (**b**) ~60 nm, (**c**) ~25 nm, (**d**) ~15 nm, (**e**), and (**f**) ~10 nm FeF_2_ NRs. (**g**) Powder XRD patterns of ~120 nm and ~25 nm FeF_2_ NRs (compared with reference PDF 045–1062) and ~10 nm FeF_2_ NPs with an asterisk indicating a minor impurity of ~10 nm FeF_2_ NRs. The impurity can be attributed to the iron oxide (magnetite) layer being present at the surface FeF_2_ NRs. (**h**) HRTEM image of ~25 nm FeF_2_ NR. (**i**–**j**) TEM images of MnF_2_ NRs synthesized in TOP (**i**) and TOPO (**j**). (**k**) TEM image of CoF_2_ NRs synthesized in TOPO.

Monodisperse MF_2_ (M = Fe, Mn, and Co) NRs were obtained by thermolysis of corresponding TMTFAs [“Fe_3_OTFA”, “Mn(TFA)_2_”, and “Co(TFA)_2_”] at temperatures of 250–320 °C with the use of TOP or TOPO as solvents (Fig. 1, see methods and Tables S1–S3 for reaction conditions). As indicated by Blake *et al*.^43^, the thermal decomposition of trifluoroacetic anions (TFA^−^) proceeds via decarboxylation, formation of the trifluoromethyl anion (CF_3_^−^), and its subsequent dissociation into a fluoride ion (F^−^) and difluoromethylene (CF_2_). Then, F^−^ can couple with a metal ion, resulting in the formation of the corresponding metal fluoride. When the decomposition is performed in a suitable solvent and in the presence of surfactant molecules, the resulting fluorides can adopt the form of uniform colloidal NRs (Fig. 2). In our experiments, the use of TOP and TOPO as solvents was essential for preparing monodisperse MF_2_ NRs because these act as neutral L-type ligands^44^, which coordinate to Lewis acidic surfaces. This mechanism might explain the preferred rod morphology of the MF_2_ NRs; *i.e*. growth in the [001] direction of the rutile-type crystal structure. The growth in other directions is likely prohibited by TOP/TOPO molecules covering the Lewis acidic, metal-rich (010) and (100) facets of MF_2_. TOPO likely binds more strongly to the transition metals than does TOP owing to its highly polarized phosphor oxygen bond. Generally, compared with other solvents, such as long-chain alkanes/alkenes, nitrogen and sulfur containing solvents (see example for FeF_2_ NRs, Figure S3), TOP and TOPO are markedly better solvents for growing MF_2_ NRs.

Highly crystalline FeF_2_ NRs were obtained by heating the “Fe_3_OTFA” precursor solution in TOP solvent to 320 °C (Fig. 1a). The length of the FeF_2_ NRs was tunable from 10 to 200 nm by addition of OA (Fig. 2a–f, Table S1), presumably because of its stronger bonding to the transition metal-rich facets, such as (010) and (100), than that of TOP solvent. At a low OA content, we also observed twinned, boomerang-shaped FeF_2_ NRs (Fig. 2c). The heating rate also affected the length of the FeF_2_ NRs, yielding longer NRs at lower heating rates.

As illustrated in Fig. 2g,h and S4a,b, the powder X-ray diffraction (XRD), high-resolution transmission electron microscope (HRTEM) images, and selected area electron diffraction (SAED) results confirmed the formation of highly crystalline FeF_2_ NRs having a tetragonal rutile-type crystal structure with the space group *P*4_2_/*mnm* (a = 4.7035 Å, c = 3.3056 Å, V = 73.13 Å^3^, PDF 045–1062). The FeF_2_ NRs showed preferred orientation in the powder XRD pattern (Fig. 2g), as indicated by narrower (101) and (002) peaks in comparison with the (110), (111), and (211) reflections; the former being parallel to the growth direction of FeF_2_ NRs. Additionally, we the (101) and (002) peaks of the FeF_2_ NRs were more intense than those of bulk FeF_2_ owing to alignment of the NRs. As the length of the FeF_2_ NRs decreased, the preferential orientation and texture effects disappeared. We note that TOP is known to be an efficient phosphor source and thus might contaminate FeF_2_ NRs. From EDX measurements of the FeF_2_ NRs (Figure S4c), only a tiny amount of phosphorus was detected.

**Figure 3:**
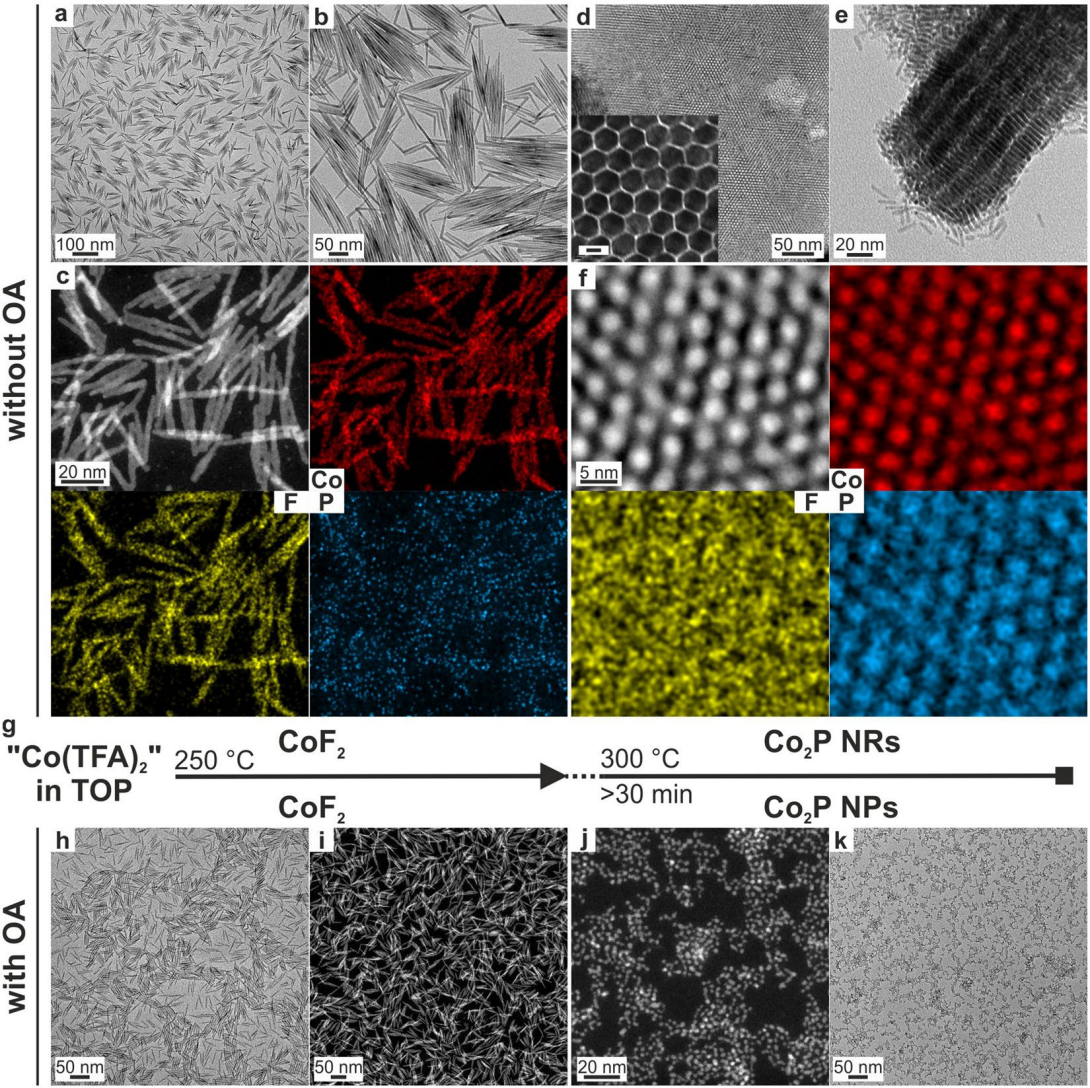
(**a**–**h**) TEM and HAADF-STEM images of the CoF_2_ NRs (**a**–**c**) and Co_2_P NRs (**d**–**f**) synthesized without OA [inset: HRTEM image of Co_2_P NRs (scale bar = 4 nm)]. For HAADF-STEM elemental mapping, the following color code was used: cobalt (red), fluorine (yellow) and phosphorous (blue). (**g**) Synthetic scheme presenting the reaction conditions for obtaining CoF_2_ and Co_2_P NRs and Co_2_P NPs. (**h**–**k**) TEM images of CoF_2_ NRs (**h**,**i**) and Co_2_P NPs (**j**,**k**) synthesized with OA.

A similar synthetic procedure to that used for FeF_2_ NRs was applied to MnF_2_ and CoF_2_ based on the “Mn(TFA)_2_” and “Co(TFA)_2_” precursors, respectively. However, we found slightly different behaviors. First, the choice of the solvent had different effects on the morphology (Fig. 1b, Table S2): 25–35 nm long MnF_2_ NRs formed in TOP (Fig. 2i) and 15–20 nm long MnF_2_ NRs formed in TOPO (Fig. 2j). In both solvents, in addition to a tetragonal phase of MnF_2_ (space group *P*4_2_/*mnm*, a = 4.8734 Å, c = 3.3099 Å, V = 78.61 Å^3^, PDF 075–1717), we observed a high-temperature orthorhombic phase (space group *Pbcn*; a = 4.96 Å, b = 5.8 Å, c = 5.359 Å, V = 154.17 Å^3^, PDF 017–0864) (Figures S5, S6); albeit the occurrence of this phase was much more pronounced in the TOPO solvent. EDX measurements of MnF_2_ NRs synthesized in TOP and TOPO revealed no contamination by phosphorus (Figure S7). The upper temperature of thermolysis was limited to *ca*. 250 °C because the MnF_2_ NRs agglomerated into large, cubic particles (~500 nm) at higher temperatures (Figure S8). For CoF_2_, we synthesized highly crystalline CoF_2_ NRs in both TOPO (Fig. 2k, Table S3) and TOP solvents (Fig. 3h,i, and Table S3) by thermolysis of “Co(TFA)_2_” at 300 and 250 °C, respectively. The CoF_2_ NRs were characterized by tetragonal-, rutile-type crystal structure types (space group *P*4_2_/*mnm*; a = 4.7106 Å, c = 3.1691 Å, V = 70.32 Å;^3^ PDF 033–0417, see Figures S9a,b, S10a). Only a small amount of phosphorous was detected in EDX of CoF_2_ NRs (Figures S9c, S10b). However, at higher temperatures, thermolysis of “Co(TFA)_2_” in the presence of TOP, which acts as a phosphor source, yielded Co_2_P NRs, as discussed in the next section.

### Synthesis of metal phosphides NRs/NPs

We also found that “Co(TFA)_2_” might also be used as a precursor for synthesizing cobalt phosphides, when combined with the solvent TOP as a phosphorous source and a solvent with heating to 300 °C (Fig. 1c). TEM analysis and EDX mapping (Fig. 3) point to the following formation mechanism (Fig. 3g): First, “Co(TFA)_2_” starts to decompose at 250 °C forming CoF_2_ NRs (Figs 3a–c and S10), which then react with TOP at 300 °C. At this point, we observed a mixture of both cobalt fluoride and cobalt phosphide (Figure S11a,b), followed by the formation of phase-pure 20 nm long cobalt phosphide NRs after 90 min (Fig. 3d–f). The obtained NRs assembled in large 3D clusters owing to their hexagonal shape and narrow size distribution. Our XRD (Figure S12) measurements indicated that the cobalt phosphide NRs crystallized in two different phases: Co_2_P as the main phase with some CoP, both having the same orthorhombic structure (space group *Pnam*; for Co_2_P: a = 5.6465 Å, b = 6.6099 Å, c = 3.513 Å, PDF 032–0306; for CoP: a = 5.077 Å, b = 3.281 Å, c = 5.587 Å, PDF 029–0497).

When OA was added to the reaction mixture of “Co(TFA)_2_” in TOP, ultra-small 3 nm cobalt phosphide NPs were obtained at 300 °C after 2 h (Fig. 3j,k). The thermolysis of “Co(TFA)_2_” in TOP with OA for a short reaction time of 0–10 min yielded the mixture of cobalt fluoride and phosphide NRs/NPs (Figure S11c,d). Our XRD and SAED measurements showed that the obtained NPs were amorphous. In addition, small crystalline regions were also visible from high-angle annular dark-field scanning transmission electron microscopy (HAADF-STEM) images (Figure S13). In accordance with the EDX measurements (Figure S13b), the chemical composition of the cobalt phosphide NPs, denoted as Co_2_P, corresponded to a 2:1 Co:P molar ratio.

We note that the cobalt phosphides have attracted considerable attention over the last three years for their ability to act as a bifunctional electrocatalyst for hydrogen^45–51^ and oxygen reduction/evolution reactions^52–57^ with low overpotentials used for water splitting^58–64^. These compounds are also used to catalyze substitution reactions of functional groups by hydrogen^65–69^, for heavy metal removal^70,71^, and for hydrogenation of CO^72^. The synthesis of Co_2_P NRs/NPs based on “Co(TFA)_2_” as a precursor has not been reported. Importantly, our synthesis of Co_2_P NRs/NPs yielded a much narrower size distribution than that achieved in NPs synthesized with Co(acac)_2_^47,57,73–75^, ε-Co NPs^76^, and others precursors^77–85^.

### Synthesis of FeF_3_ NRs

**Figure 4:**
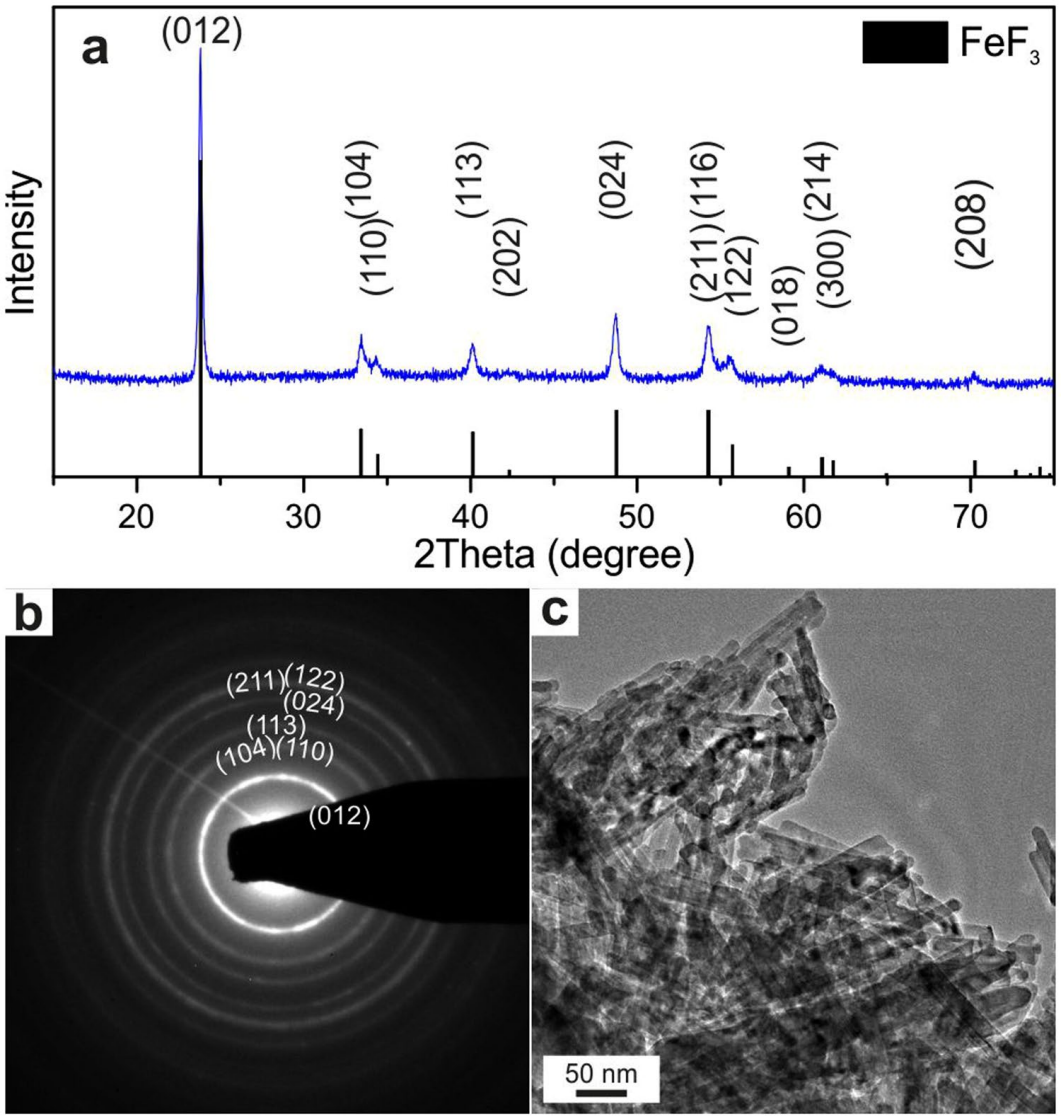
Characterization of FeF_3_ NRs. Powder XRD pattern (**a**), SAED image (**b**), and TEM image (**c**) of FeF_3_ NRs.

We tested fluorination of FeF_2_ NRs with fluorine gas as a route to FeF_3_ NRs. Briefly, ~120 nm long FeF_2_ NRs were fluorinated in a powder form at 500 °C with an Ar/F_2_ gas mixture for 30 min in an aluminum tube (see Experimental Section for details). Our XRD and SAED measurements showed that FeF_2_ NRs were fully converted to FeF_3_ NRs (Fig. 4a,b); the latter crystallized in a ReO_3_-type structure of FeF_3_ (R-FeF_3_) with the space group *P*-3*c* (167) (a = 5.2 Å and c = 13.323 Å, V = 312 Å^3^, PDF 033–0647). The rod shape of the initial FeF_2_ NRs was retained. However, the FeF_3_ NRs were four times as large as the FeF_2_ NRs (Fig. 4c) owing to the volume difference between the crystal structures of the FeF_2_ and R-FeF_3_. Additionally, agglomeration of R-FeF_3_ NRs was observed during fluorination caused by concomitant ligand removal from the FeF_2_ NRs surface. We note that the fluorine gas flow had considerable effects on the complete fluorination of the FeF_2_ NRs. The temperature was also an important parameter. The formation of R-FeF_3_ started at a low temperature of 350 °C; however, highly crystalline and phase-pure R-FeF_3_ NRs were only obtained at 500 °C.

### Electrochemical performance of FeF2 and FeF3 NRs

For the electrochemical measurements, we prepared electrodes by mixing a powder of FeF_2_ or FeF_3_ NRs with carbon additives [graphene oxide (GO) and/or carbon black (CB)], polyvinylidene fluoride (PVdF), and *N*-methylpyrrolidone (NMP) solvent (see Experimental Section for details). First, the FeF_2_ or FeF_3_ NRs were dry ball-milled with GO/CB or CB. Then the resulting powder was subjected to one more step of ball-milling with the pVdF polymer as a binder and NMP as a solvent, followed by casting the slurry onto Al foil. Afterward, the electrodes were dried under vacuum at 80 °C for 24 h.

**Figure 5:**
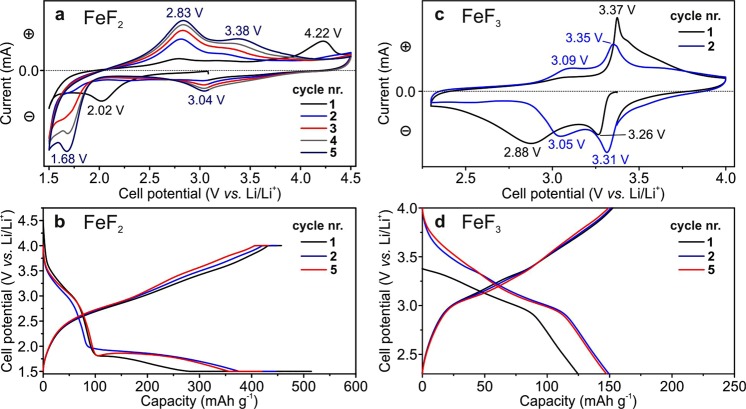
Electrochemical characterization of FeF_2_ and FeF_3_ NRs vs. lithium. Cycling voltammetry of FeF_2_ (**a**) and FeF_3_ (**c**) NRs at scanning speeds of 0.2 and 0.1 mV s^−1^, respectively. Galvanostatic charge–discharge curves of FeF_2_ (**b**) and FeF_3_ NRs (d) for 1^st^, 2^nd^, and 5^th^ cycles at a current densities of 200 and 50 mA g^−1^, respectively.

We tested the FeF_2_ NRs *vs*. Li^+^/Li in the voltage range of 1.5–4.0 V, which includes both conversion and insertion regions. From the CV curves (Fig. 5a), lithiation of electrodes composed of FeF_2_ NRs during the first discharge was characterized by the appearance of a peak at 2 V *vs*. Li^+^/Li associated with reduction of graphene oxide followed by the intensity increase of the negative current at 1.5 V *vs*. Li^+^/Li indicating a conversion process of FeF_2_ NRs (FeF_2_ + 2Li^+^  + 2e^−^ → Fe + 2LiF). The formation of Fe and LiF during lithiation of FeF_2_ has been reported in numerous studies based on *in situ*^86^ and *ex situ*^19,87–89^ methods. In the reverse scan, the FeF_2_ electrode displayed two peaks at 2.8 and 3.4 V *vs*. Li^+^/Li, which are associated with the formation of Li_0.5_FeF_3_ and Li_0.25_FeF_3_ phases. The third peak at a higher potential of 4.2 V was related to a pronounced and unknown irreversible reaction. Upon further cathodic cycling, a peak at 3.0 V *vs*. Li^+^/Li appeared, which we attributed to the formation of a Li_0.25_FeF_3_ phase. We note that the intensity increase of CV curves during the initial cycles might be attributed to restructuring processes in the electrode caused by the formation of metallic Fe, which lowers the resistivity of the electrodes. As shown in Fig. 5b, the discharge voltage profiles of the FeF_2_ NRs were similar to CV curves representing distinct insertion (3.4–2.6 V *vs*. Li^+^/Li) and conversion (1.5–2.0 V *vs*. Li^+^/Li) reactions. However, upon charging, the galvanostatic curves were rather smooth, which suggested a slow gradual de-lithiation processes.

Figure 5c shows cyclic voltammetry curves of the electrodes composed of FeF_3_ NRs at a scan rate of 0.1 mV s^−1^. In the first cathodic cycle (lithiation step), the first two peaks appear at approximately 3.26 and 3.88 V *vs*. Li^+^/Li. We attribute these features to electrochemical lithiation of the FeF_3_ NRs. We note that the mechanism of Li^+^ intercalation into FeF_3_ has been examined experimentally and theoretically; however, many of the details remain unclear. It is believed that the initial ReO_3_-type structure of FeF_3_ transforms into a tri-rutile-like structure with a composition of Li_0.25_FeF_3_ and Li_0.5_FeF_3_, which is followed by the formation of FeF_2_ and LiF (for an overall one-electron process) upon further reduction. In the reverse scan, two distinct reversible de-lithiation reactions of FeF_3_ NRs are observed suggesting the existence of two prevailing intermediate states of de-lithiated FeF_3_ (Li_0.5_FeF_3_ at *ca*. 3.1 V and Li_0.25_FeF_3_ at *ca*. 3.3 V), based on studies by Doe *et al*.^90^, Yamakawa *et al*.^91^, and Li *et al*.^9^ Fig. 5d shows the typical voltage profiles of the Li-ion half-cells based on FeF_3_ NRs as an active material at a current density of 50 mA g^−1^. The shape of the voltage profiles and the cyclic voltammetry (CV) curves, were relatively sharp with a low polarization compared with previously reported data on FeF_3_ cathodes^13,92,93^. These results point to an intermittent mechanism of lithiation/de-lithiation of the FeF_3_ NRs through the formation of intermediate Li_0.5_FeF_3_ and Li_0.25_FeF_3_ phases. The galvanostatic measurements of FeF_2_ and FeF_3_ NRs are presented in Figures S14 and S15, respectively.

## Conclusions

This work presents a simple synthetic route to prepare high-quality Fe, Mn, and Co difluoride NRs via thermolysis of transition metal (M = Fe, Mn, and Co) trifluoroacetate complexes in TOP and TOPO solvents. We show that the use of TOP or TOPO solvents is essential for synthesizing monodisperse MF_2_ NRs, which act as neutral L-type ligands that coordinate to Lewis acidic surfaces leading to the preferred rod morphology (i.e., growth in the [001] direction). The length of the FeF_2_ NRs was tunable by the addition of oleic acid owing to its stronger bonding to transition metal-rich facets such as (010) and (100). A bottom-up synthesis of high crystalline phase-pure FeF_3_ NRs by fluorination of our FeF_2_ NRs by fluorine gas is also reported.

We show that the cobalt trifluoroacetate complex can be thermally decomposed in a TOP solvent system to yield cobalt phosphide NRs/NPs. The reaction mechanism includes: thermolysis of “Co(TFA)_2_” with the formation of CoF_2_ NRs following their reaction with TOP at higher temperatures of 300 °C leading to highly monodisperse 20 nm long Co_2_P NRs or 3 nm Co_2_P NPs (in the presence of oleic acid).

We also assessed the electrochemical storage of Li^+^ ions in FeF_2_ and FeF_3_ NRs. Our studies are currently underway towards optimization of electrodes composed of FeF_2_ and FeF_3_ NRs (*e.g*., carbon encapsulation) and their electrochemical performance (choice of voltage intervals, and electrolytes).

## Supplementary information

R1_Supporting Information_revised
