# Supplementary material for: Transition metal trifluoroacetates (M = Fe, Co, Mn) as precursors for uniform colloidal metal difluoride and phosphide nanoparticles
Source: Sci Rep. 2019 Apr 29;9:6613. doi: 10.1038/s41598-019-43018-8 (PMC6488600; doi:10.1038/s41598-019-43018-8)
Supplement: Supplementary file 1 — R1_Supporting Information_revised [file 41598_2019_43018_MOESM1_ESM.docx]

*Electronic Supplementary Information for*

Transition metal trifluoroacetates (M = Fe, Co, Mn) as precursors for uniform colloidal metal difluoride and phosphide nanoparticles

Christoph P. Guntlin,^†,§^ Kostiantyn V. Kravchyk, ^†,§^ Rolf Erni,^‡^ and Maksym V. Kovalenko^*,†,§^

^†^Department of Chemistry and Applied Biosciences, ETH Zürich - Laboratory for Inorganic Chemistry, Vladimir Prelog Weg 1, CH-8093 Zürich, Switzerland

^§^Laboratory for Thin Films and Photovoltaics, Empa – Swiss Federal Laboratories for Materials Science and Technology, Überlandstrasse 129, CH-8600 Dübendorf, Switzerland

^‡^Electron Microscopy Center, Empa – Swiss Federal Laboratories for Materials Science and Technology, Überlandstrasse 129, CH-8600 Dübendorf, Switzerland

KEYWORDS: FeF_2_, CoF_2_, MnF_2_, FeF_3_, Co_2_P, nanorods, nanoparticles, Li-ion battery, cathode.

Corresponding Author:

*E-mail: [mvkovalenko@ethz.ch](mailto:mvkovalenko@ethz.ch)

**Table S1.** The reaction conditions for obtaining FeF_2_ NRs using "Fe_3_OTFA" precursor and their characterization.

| Solvent | OA (mmol) | Heating rate (°C min^-1^) | Temperature  (°C) | Reaction time  (min) | Mean size (nm) | Shape | Product | Phase | Color |
| --- | --- | --- | --- | --- | --- | --- | --- | --- | --- |
| TOP | no | 6 | 320 | 0 | 200 | NRs | FeF_2_ | rutile | black |
|  | no | 18 |  | 0 | 120 |  |  |  |  |
|  | 0.75 | 6 |  | 0 | 60 |  |  |  |  |
|  | 1.5 | 18 |  | 20 | 25 |  |  |  |  |
|  | 2.25 | 6 |  | 0 | 15 |  |  |  |  |
|  | 3 | 6 |  | 0 | 10 |  |  |  |  |

**Table S2.** The reaction conditions for obtaining MnF_2_ NRs using "Mn(TFA)_2_" precursor and their characterization. The weight ratio (%) between the tetragonal and orthorhombic phase was refined from Rietveld analysis (see Figure S4).

| Solvent | OA (ml) | Heating rate  (°C min^-1^) | Temperature  (°C) | Reaction time  (min) | Mean size (nm) | Shape | Product | Phase  (wt. %) | Color |
| --- | --- | --- | --- | --- | --- | --- | --- | --- | --- |
| TOPO | no | 6 | 250 | 0 | 20 | NRs | MnF_2_ | tetragonal (49.3)  orthorhombic (50.7) | colorless |
| TOP | no | 6 | 250 | 0 | 35 | NRs | MnF_2_ | tetragonal (62.2)  orthorhombic (37.8) | brown |

**Table S3.** The reaction conditions for obtaining CoF_2_ NRs, Co_2_P/CoP NRs and “CoP” NPs using "Co(TFA)_2_" precursor and their characterization.

| Solvent | OA (ml) | Heating rate  (°C min^-1^) | Temperature  (°C) | Reaction time  (min) | Mean size (nm) | Shape | Product | Phase | Color |
| --- | --- | --- | --- | --- | --- | --- | --- | --- | --- |
| TOPO | 0.5 | 6 | 300 | 20 | 35 | NRs | CoF_2_ | rutile | mangenta |
| TOP | no | 6 | 250-300 | *in situ* | 70-250 | NRs | CoF_2_ | rutile | mangenta |
|  | no | 6 | 300 | 120 | 40 | NRs | Co_2_P | ortho-rombic | black |
|  | 0.5 | 6 | 250-300 | *in situ* | 45-60 | NRs | CoF_2_ | rutile | mangenta |
|  | 0.5 | 6 | 300 | 90 | 3 | NPs | Co_2_P | ortho-rombic | black |

**
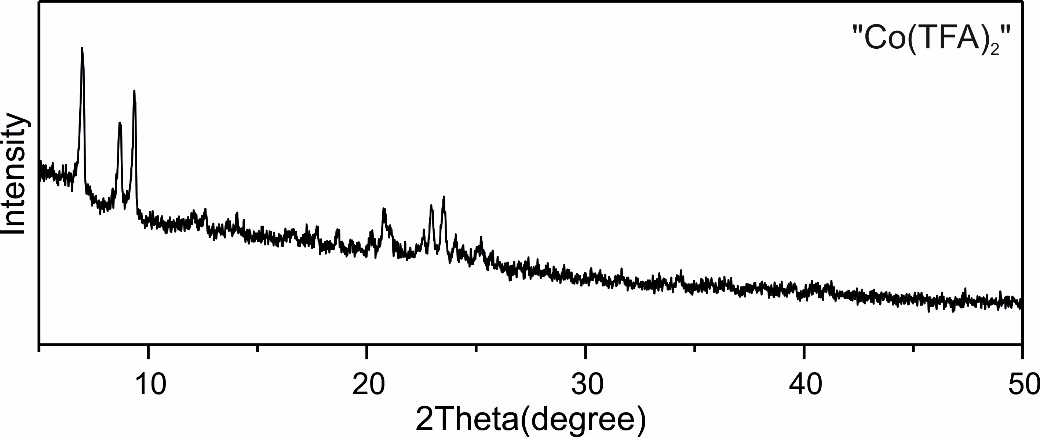
**

Fig. S1. Powder XRD pattern of “Co(TFA)_2_”.


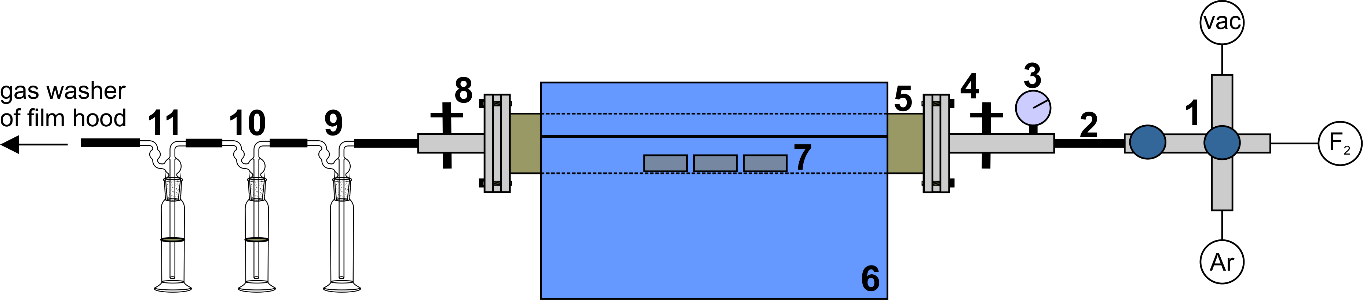


Fig. S2. Fluorination set-up. 1 is a gas valve, 2 is Teflon tube, 3 is manometer, 4 is inlet valve with stainless steel cap, 5 is Al_2_O_3_ tube, 6 is tube furnace (Across International, STF1200), 7 is Al_2_O_3_ crucibles with reactants, 8 is outlet valve with stainless steel cap, 9 is empty flask, 10 is flask with water, 11 is flask with a CaCO_3_/water solution.


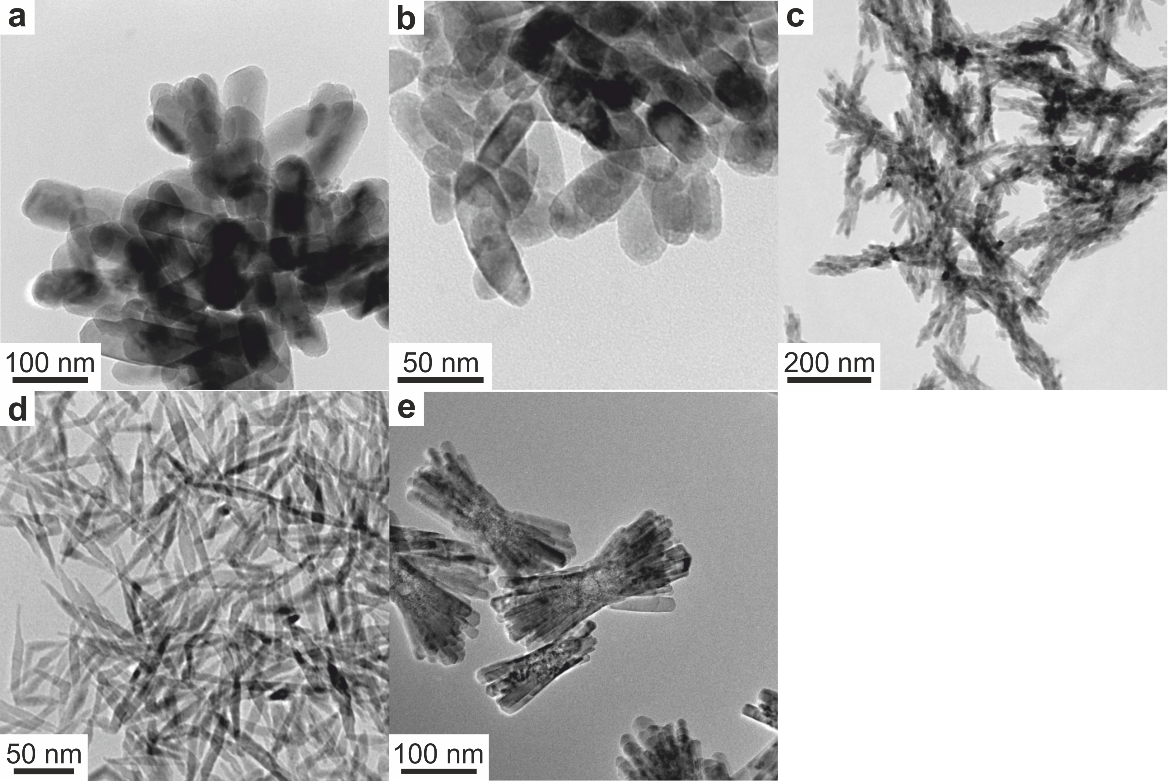


Fig. S3. TEM images of FeF_2_ NPs/NRs synthesized by the thermal decomposition of “Fe_3_OTFA” precursor in squalene (a) trioctylamine (b) sulfolane (c) tributylphosphine (d) tetramethylene sulfone (e).


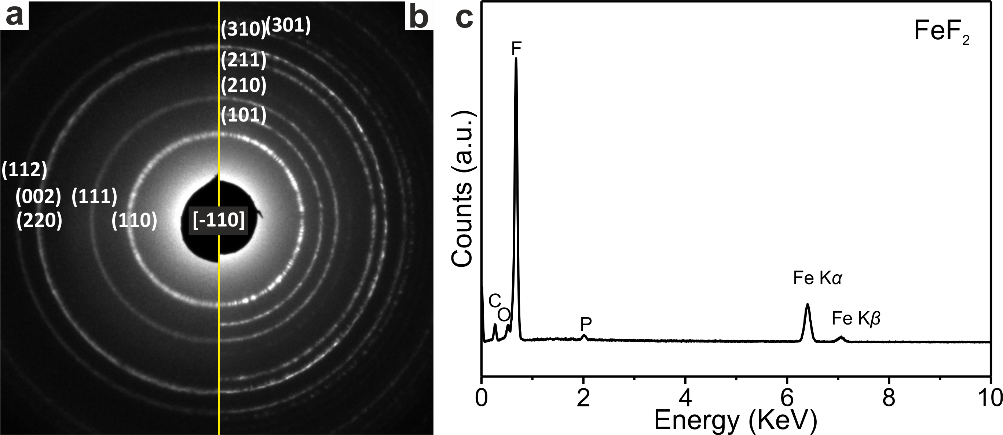


Fig. S4. SAED patterns of 120 nm FeF_2_ NRs (a) and 10 nm FeF_2_ NRs (b). 10 nm FeF_2_ NRs show preferred crystallographic orientation leading to vanished reflexions from the planes, which are not parallel to the zone axis [-110]. (c) EDX measurements of 120 nm FeF_2_ NRs (c), which are representative also for all other lengths of FeF_2_ NRs. The residual phosphorus peak is attributed to the presence of TOP as a ligand on the surface of FeF_2_ NRs.


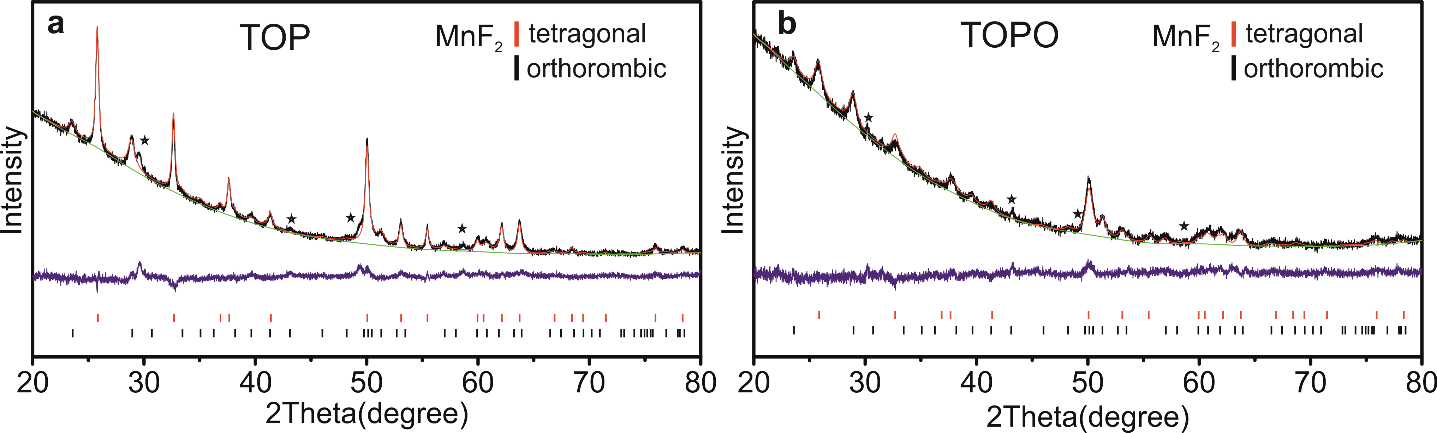


**Fig. S5.** A comparison of the experimental (black) and calculated (red) powder diffraction patterns of MnF_2_ NRs/NPs synthesized in TOP (a) and TOPO (b) together with the difference (dark purple) and the reflection positions (red and black markers) of tetragonal and orthorhombic phases, as obtained from the Rietveld refinement with GSAS-II.^1^ The refinement was performed using the following structural data: a= 4.8734 Å, c= 3.3099 Å, V=78.61 Å^3^ (PDF 075-1717) for tetragonal phase and a=4.96 Å, b=5.8 Å, c= 5.359 Å, V=154.17 Å^3^ (PDF 017-0864) for orthorhombic phase. The figure of merits were wRp = 2.85%, GOF = 1.55 for tetragonal phase and wRp = 2.63%, GOF = 1.30 for for orthorhombic phase. The following phase fractions in MnF_2_ NPs synthesized in TOP and TOPO were refined from Rietveld analysis: 62 wt. % (tetragonal)/38 wt. % (orthorhombic) in TOP and 49 wt. % (tetragonal)/51 wt. % (orthorhombic) in TOPO.


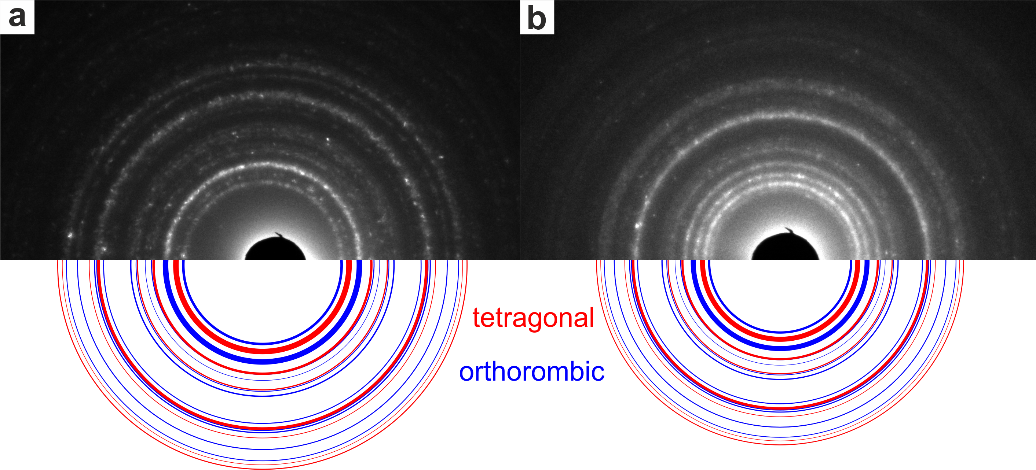


**Fig. S6.** SAED patterns of MnF_2_ NRs/NPs synthesized in TOP (a) and TOPO (b). Tetragonal (PDF 075-1717) and orthorhombic (PDF 017-0864) phases of MnF_2_ are marked by red and blue rings, respectively. The tetragonal phase in MnF_2_ NPs synthesized in TOPO appears to be dominant compared to the powder XRD studies due to the preferred orientation of the NRs on the TEM grid.


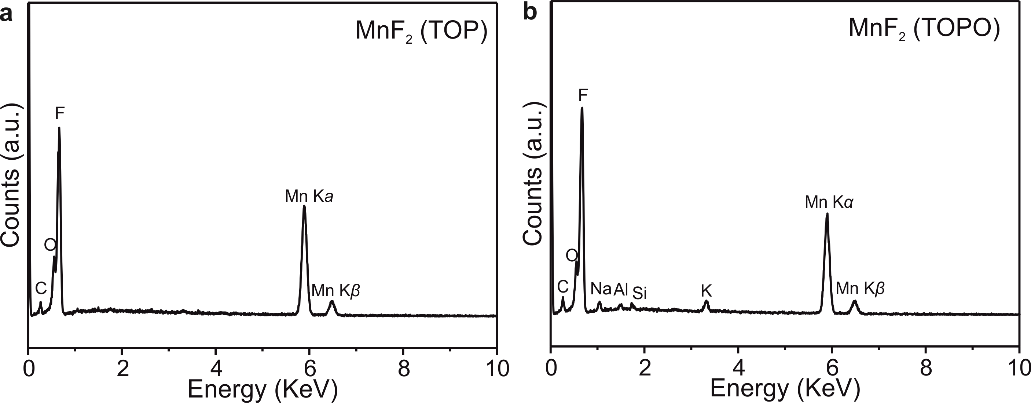


Fig. S7. EDX measurements of MnF_2_ NRs/NPs synthesized in TOP (a) and TOPO (b). Na, Al and Si impurities were detected for MnF_2_ NPs synthesized in TOPO.


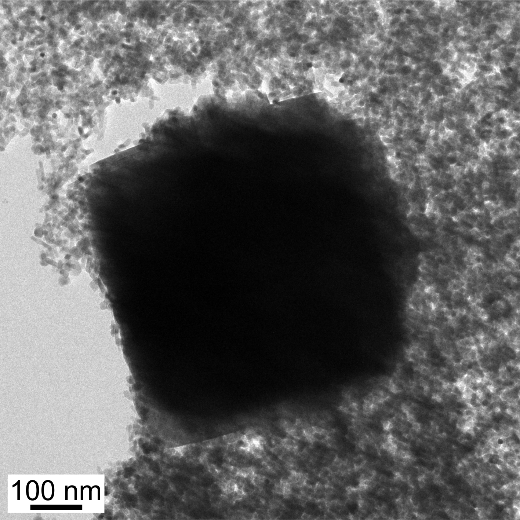


Fig. S8. TEM image of MnF_2_ NPs synthesized in TOPO at 270 °C.


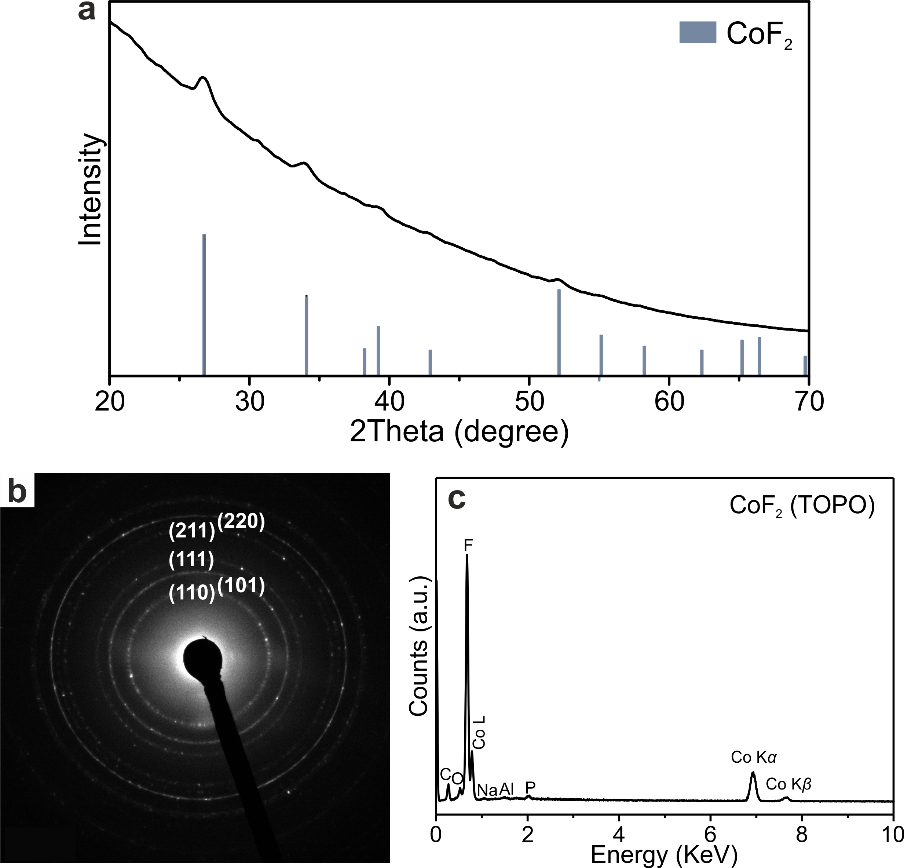


Fig. S9. Powder XRD pattern (a), SAED pattern (b) and EDX analysis (c) of CoF_2_ NRs synthesized in TOPO with the addition of OA. Stick patterns on Figure S6a represent reflections of tetragonal structure of CoF_2_ (a=4.7106 Å, c=3.1691 Å, V= 70.32 Å^3^; PDF 033-0417).


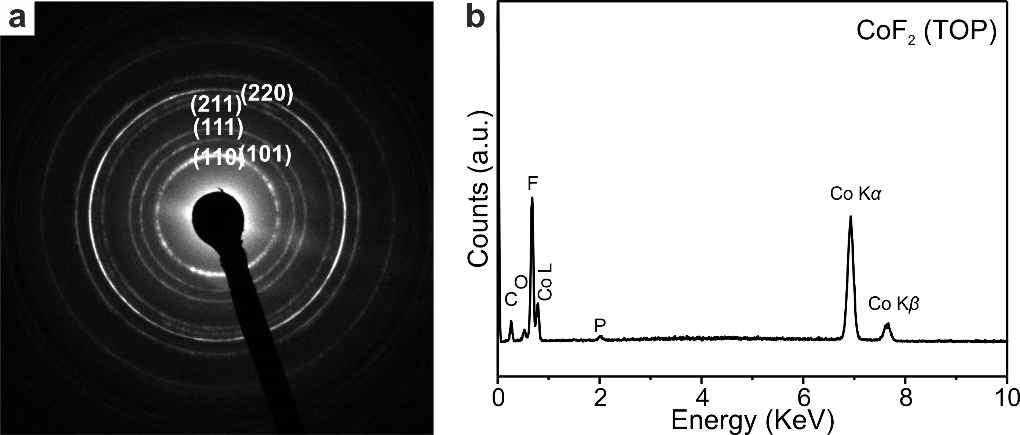


Fig. S10. SAED pattern (a) and EDX analysis (b) of CoF_2_ NRs synthesized in TOP (see Table S3 for reaction conditions, the aliquot was taken at T=250 °C).


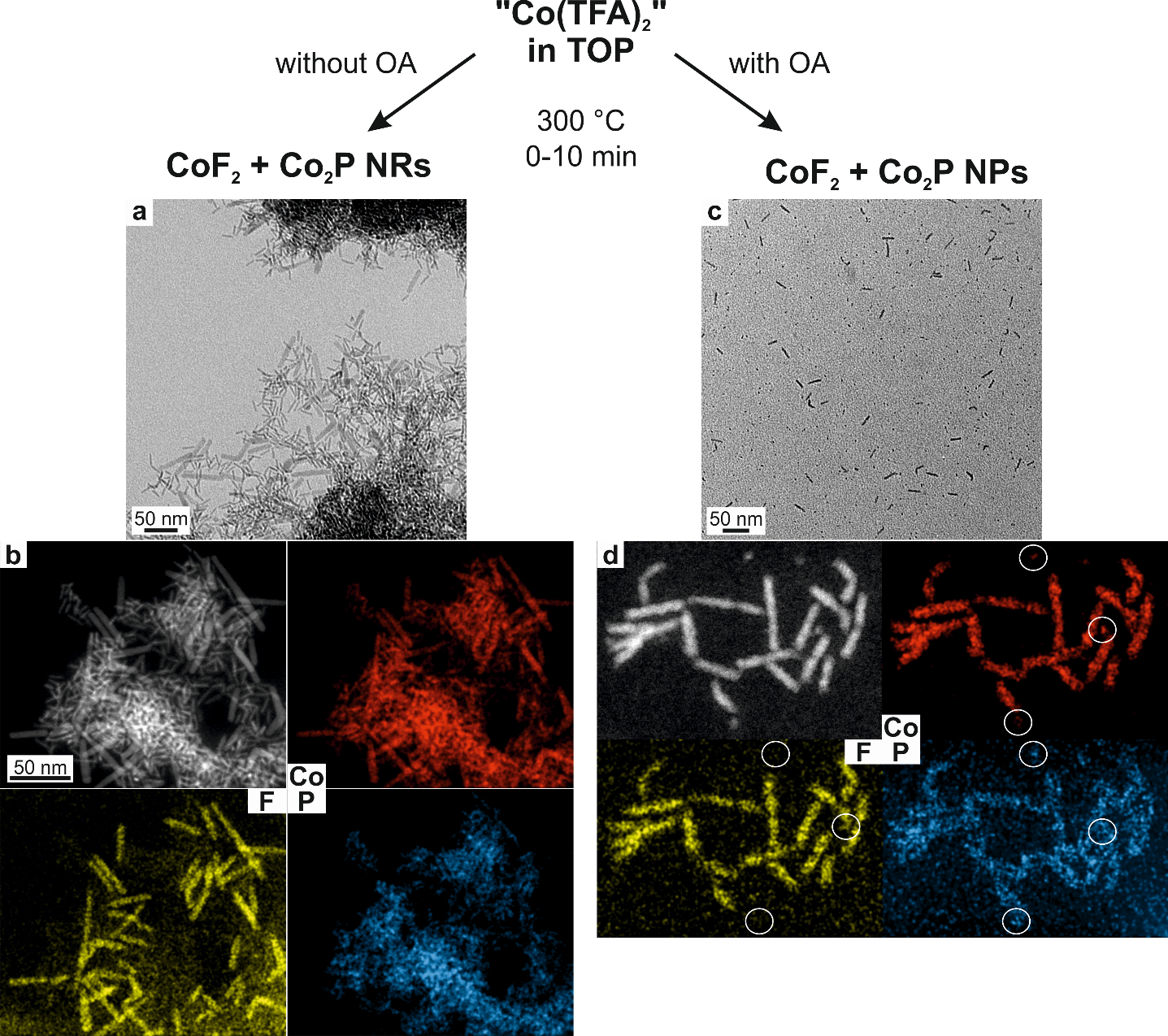


Fig. S11. TEM and HAADF STEM images of the CoF_2_ NRs + Co_2_P NPs (a,b) and CoF_2_ NRs + Co_2_P NPs (c,d) synthesized in TOP at 300 °C without and with OA, respectively (the aliquots were when the reaction solutions reached the temperature of 300 °C). For HAADF-STEM elemental mapping, the following color code was used: cobalt (red), fluorine (yellow) and phosphorous (blue). White cycles show the regions with Co_2_P NPs.


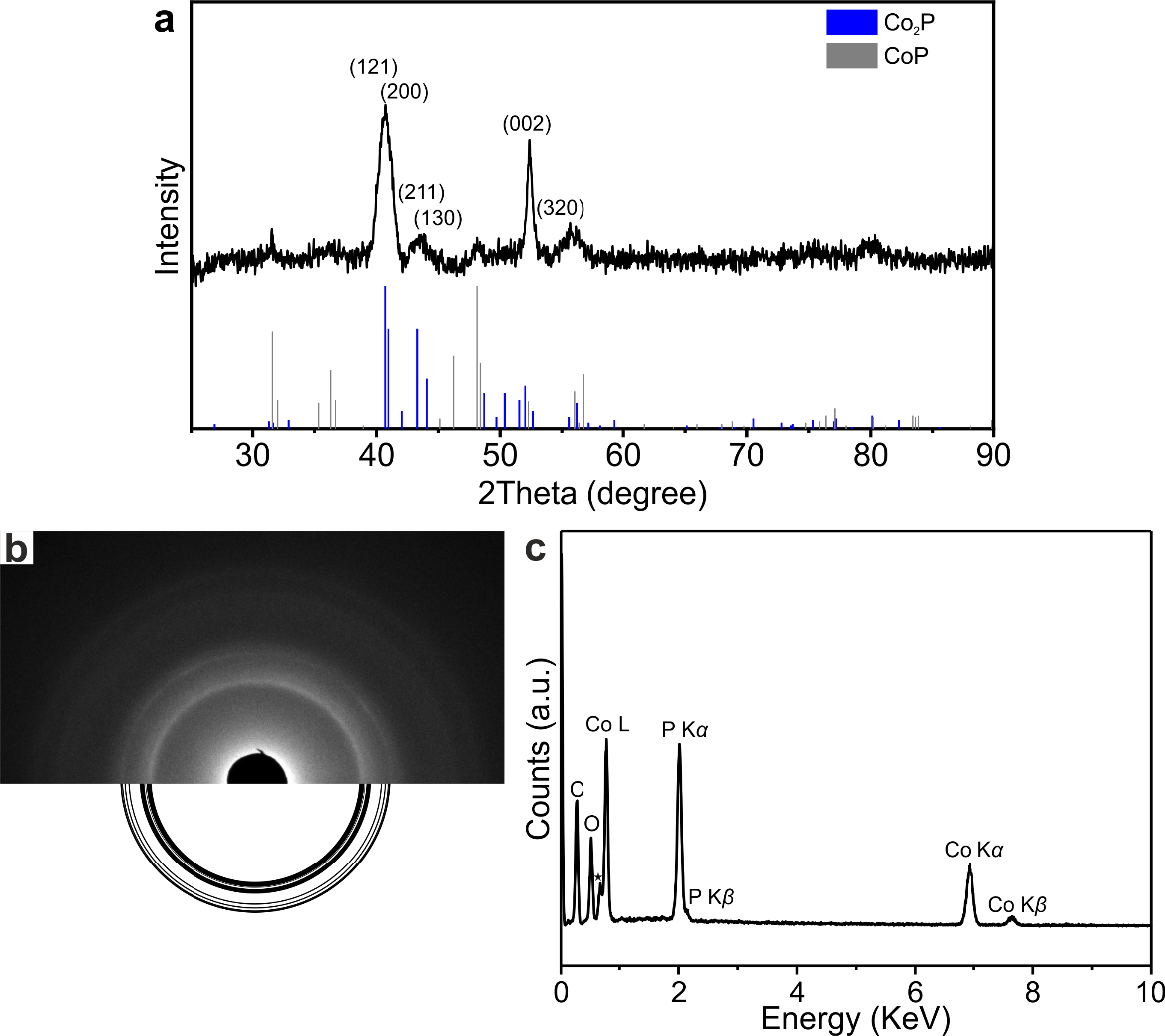


Fig. S12. Powder XRD pattern (a), SAED pattern (b) and EDX analysis (c) of Co_2_P NRs synthesized in TOP at 300 °C (reaction time = 2 h). Stick patterns in Figure S12a represent reflections of Co_2_P (PDF 032-0306)) and CoP (PDF 029-0497) phases. Due to a preferred orientation, the intensity of 211 peak appears to be lower than for the 002 peak. This observation implicates a growth direction of the rods perpendicular to the 002 plane. Co_2_P (PDF 032-0306) phase is marked by the black ring on SAED pattern (the reference pattern of CoP phase is omitted due to poor readability).


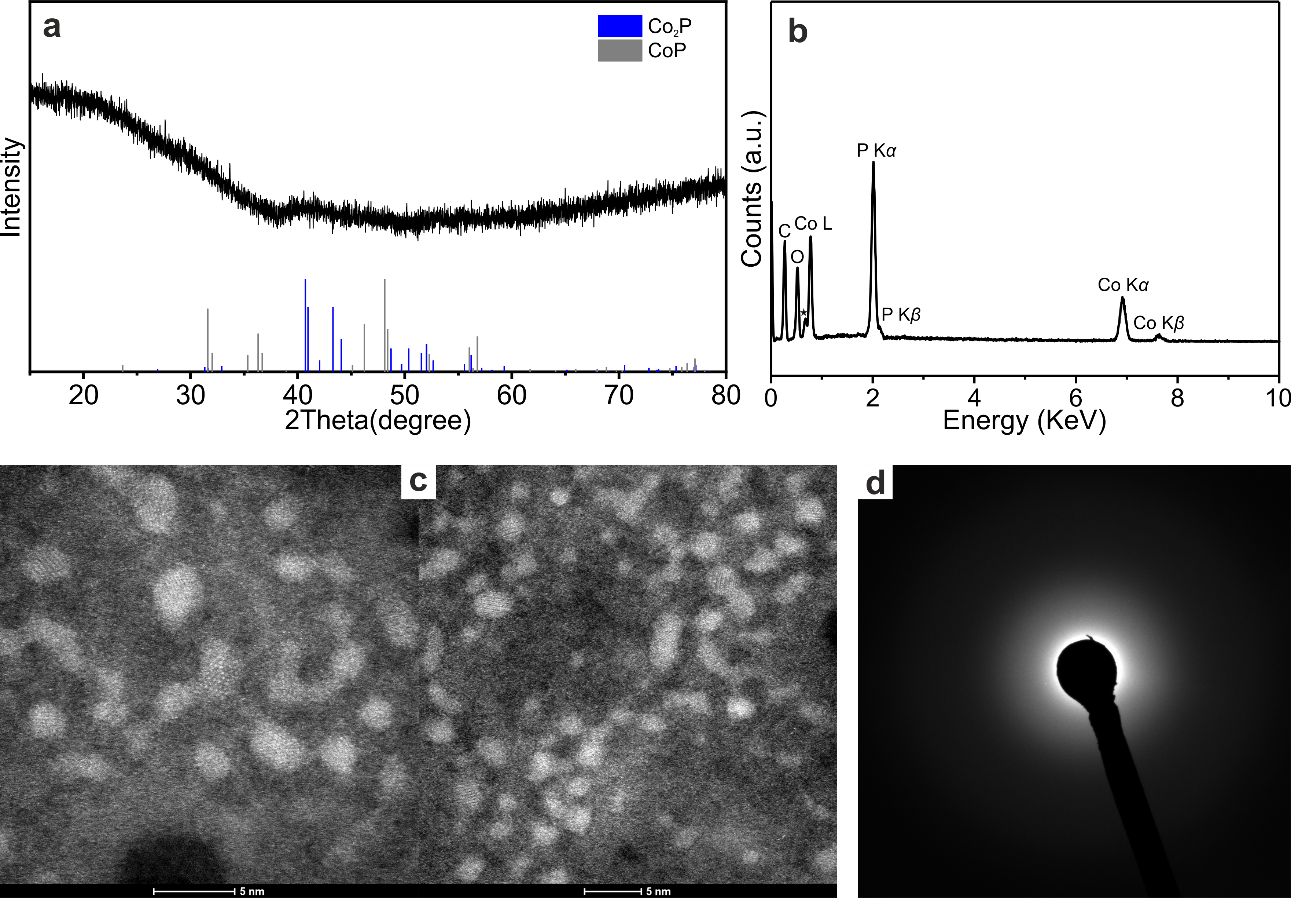


Fig. S13. Powder XRD pattern (a), EDX analysis (b), HRTEM images (c) and SAED pattern (d) of Co_2_P NPs synthesized in TOP at 300 °C (reaction time = 1.5 h) with addition of OA. Lattice fringes on HRTEM images show the crystallinity of Co_2_P NPs. Their size varies between 2-3.5 nm.


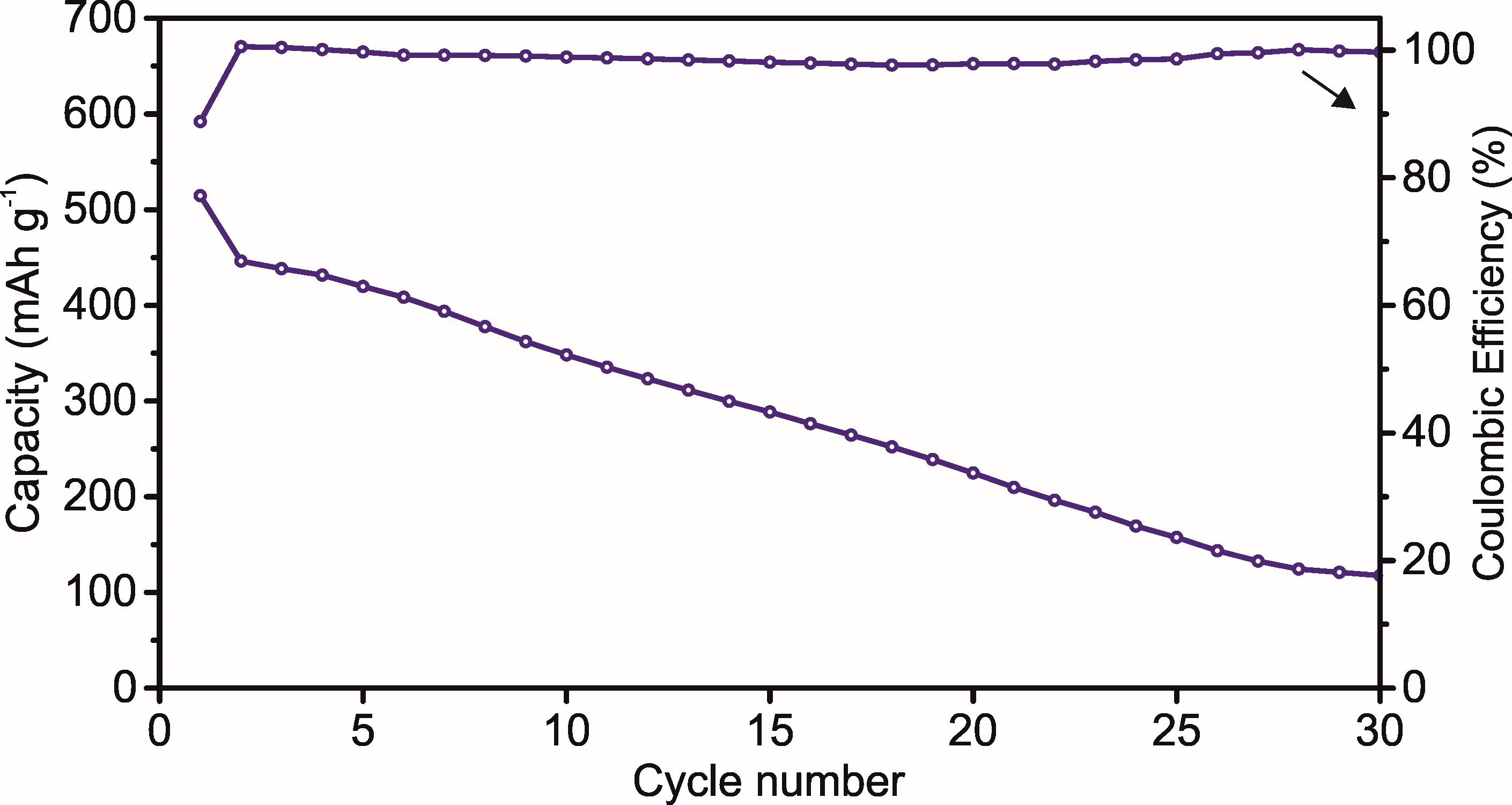


Fig. S14. Galvanostatic measurement of FeF_2_ NRs (120 nm) *vs.* lithium. The measurement was conducted between 1.5 V – 4.0 V with a current density of 200 mA g^-1^.


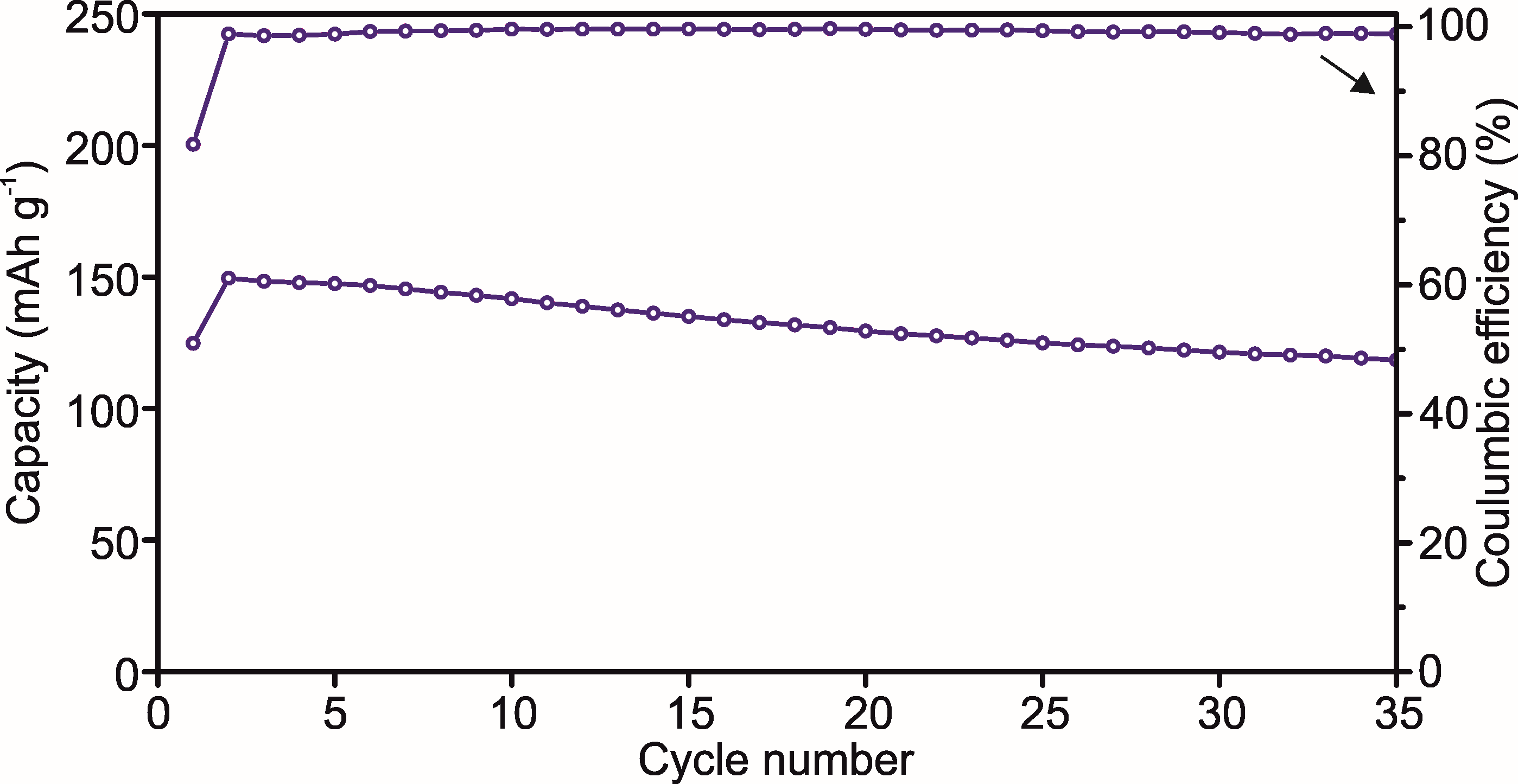


Fig. S15. Galvanostatic measurement of FeF_3_ NRs *vs.* lithium conducted between 2.3-4.0 V with a current density of 50 mA g^-1^.

**Reference**

1. Toby, B. H.; Von Dreele, R. B., GSAS-II: the genesis of a modern open-source all purpose crystallography software package. *J. Appl. Crystallogr.* **2013,** *46* (2), 544-549.
